# Intra-operative femoral head vascularity assessment: An innovative and simple technique

**DOI:** 10.4103/0019-5413.80041

**Published:** 2011

**Authors:** Vrisha Madhuri, Vivek Dutt, Kunder Samuel, Abhay Deodas Gahukamble

**Affiliations:** Department of Orthopaedics, Christian Medical College, Vellore, Tamil Nadu, India; 1 Anaesthesia, Christian Medical College, Vellore, Tamil Nadu, India

**Keywords:** Femoral head, blood flow, femoral head vascularity assessment, intraoperative, arterial transducer, safe surgical dislocation

## Abstract

**Background:**

Documentation of femoral head blood flow before, during and after head preserving surgery is important for safeguarding vascularity to the femoral head and for documentation in patients in whom the blood flow is compromised. Laser Doppler flowmetry and microsensor intracranial pressure (ICP) transducers have been used to satisfactorily depict such changes. However, these devices are expensive and not universally available in orthopedic operating rooms. We describe a new technique for the assessment of intra-operative blood flow to the femoral head. This is a technical description of a simple system utilized in eight patients to assess the femoral head vascularity using equipment available with the anesthetist.

**Materials and Methods:**

A standard epidural catheter attached to an arterial pressure transducer is introduced into the femoral head from the margin of the articular surface via a small hole drilled with a K wire. The pressure wave within the epiphysis is detected on the anesthesia monitor. Pressure within the femoral head is used as a surrogate for blood flow. The pressure and the wave form are correlated with the electrocardiogram (ECG) wave on the anesthetic machine. The technique was used in eight children with hip pathology requiring hip dislocation for documenting the hip vascularity status.

**Result:**

There was good correlation between the pressure wave and the ECG for a patient with presumed normal femoral head vascularity, whereas the pressure measurements were greatly reduced and the wave form was absent in a femoral head wih radiographic or bone scan evidence of avascular necrosis.

**Conclusion:**

This new technique is a cheap and readily accessible alternative to Laser Doppler flowmetery and ICPs monitoring probes for the assessment of blood flow to the femoral head.

## INTRODUCTION

Hip surgeries where preservation of the femoral head is required are becoming common.^1^–^4^ Pinning of the femoral neck, slipped capital femoral epiphysis and surgical dislocations carried out for impingement and other reasons require monitoring of the femoral head blood flow.^5^ In all these procedures, a documentation of femoral head blood flow before, during and after the surgery is important for safeguarding vascularity to the head, and, in patients where the blood flow is compromised it will also protect the surgeon medicolegally. Safe surgical dislocation as described by Ganz is increasingly becoming popular^1^–^4^ and is based on an understanding of the femoral head blood supply and avoids damaging this by protecting the vessels during surgery, restricting the dislocation to less than 6 hour and assessing the vascularity by observing the bleeding from the holes drilled in the femoral head. A more objective technique for assessing vascularity of the femoral head intra-operatively is desirable.

Notzli *et al*. described laser Doppler flowmetry (LDF) to monitor the circulation in the femoral head during various positions and manoeuvers.^5^ The Codman microsensor intracranial pressure (ICP) transducer has been demonstrated to satisfactorily depict the changes in the blood flow through the femoral head during various positions and when pressure is applied on the vessels to the femoral head. In specialized practices, where safe surgical dislocations are carried out routinely, commercial devices to measure head vascularity are available. These devices are expensive and are not readily available in an average orthopedic operating room.

We describe the use of an arterial transducer, commonly available with the anesthetist, for documenting the pressures in the femoral head during surgeries on the femoral head. The device can be easily assembled and is a relatively inexpensive tool to monitor blood flow. We report our observation on eight patients.

## MATERIALS AND METHODS

Between January 2010 and July 2010, eight patients who underwent surgical dislocation of the femoral head for various indications, such as Perthes disease (n=2), acute (n=2) and healed (n=2) slipped capital femoral epiphysis (SCFE), osteoid osteoma (n=1) and osteochondroma of the femoral neck (n=1), had an intra-operative femoral head vascularity assessment. The intra-operative assessment was validated by a post-operative technetium bone scan in those with acute SCFE because of the high risk of avascualrity due to the primary pathology. The patients were followed-up at 2 weeks, 2 months, 3 months, 6 months and longer periods with radiographs depending on the underlying pathology.

**Figure 1:**
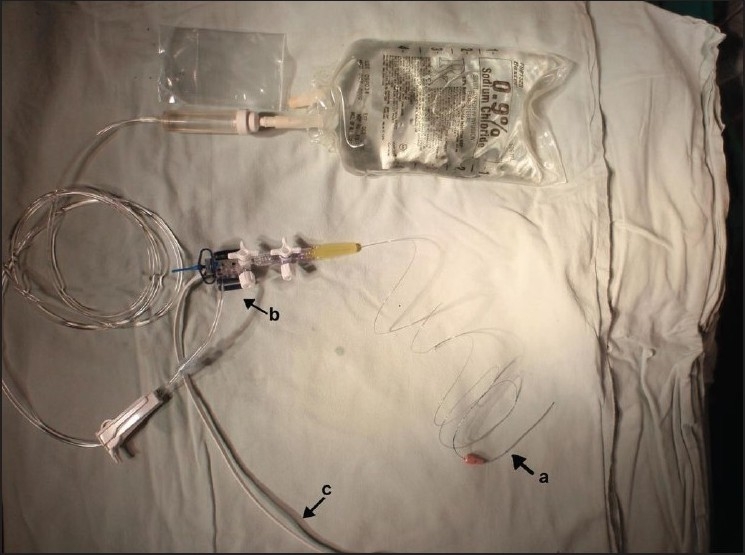
The assembly consisting of a 16 gauge epidural catheter (Braun) (a), Luer lock connector (b) and pressure transducer kit (c) that is connected to a Philips IntelliVue MP20 anesthesia monitor seen in Figure 3

The intra-operative technique was standardized per-operatively and then carried out in a similar manner in all the patients. This consisted of four steps: (1) exposure of the femoral head and dislocation preserving the blood supply usually by the safe surgical dislocation technique of Ganz, (2) assembling of the device by connecting the epidural catheter to the arterial transducer that is connected to the monitor as explained below [Figure 1], (3) a hole is drilled with a K wire on the anterolateral edge of the femoral head and insertion of the epidural catheter and (4) pressure wave compared with the ECG trace on the monitor.

The assembly requires a 16 gauge epidural catheter (B.Braun Melsungen AG), a Luer lock connector, a pressure transducer kit (B L Lifesciences Greater Noida, India) and an anesthesia monitor (Philips IntelliVue MP20 Boeblingen, Germany). The 16 gauge epidural catheter is connected to a pressure transducer kit via a Luer lock connector. The bacterial filter provided with the kit is excluded as it would dampen the pressure trace. The pressure transducer has a sterile flushing system using normal saline without heparin. The entire kit is primed with saline so that it is free of air bubbles [Figure 1].

**Figure 2:**
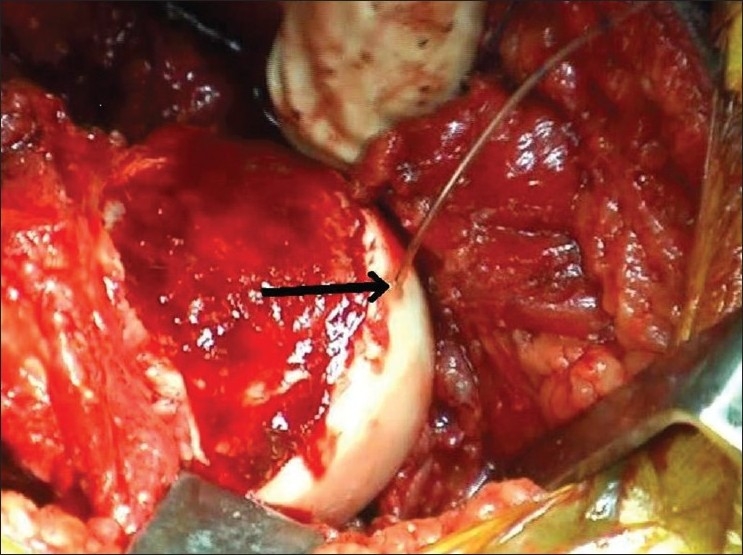
The epidural catheter is inserted into the hole drilled into the anterolateral femoral head

A hole with a 1.8 mm K wire is drilled at the anterolateral edge of the articular surface deep into the proximal femoral epiphysis. In a vascular head, the blood flows out from this after the drilling. The catheter is inserted in this pre-drilled hole [Figure 2]. One should take care to ensure that the epidural catheter is inserted in the head beyond its lateral holes at the tip. In a smaller child, the tip can be shortened by cutting close to the lateral perforations in the epidural catheter. A modification of the technique involves using the arterial line with an IV canula. Care has to be taken to avoid kinking of the canula. When femoral head is vascular, a pressure wave with its peak and rhythm corresponding to the QRS complex of the ECG trace in the monitor is seen. The pressure in our system varied from 39 to 52 mm Hg. The peak of the pressure wave also corresponded to the peak of the pulse oxymeter wave. The avascular heads showed no wave form and very low pressures, varying from 0 to 2 mm Hg.

In children undergoing the capital realignment technique or surgical dislocation for any surgical procedure, the technique allowed intra-operative monitoring of the head blood flow. In those with suspected avascular necrosis and no flow, such as in acute SCFE, it documented the lack of blood flow to the head, protecting the surgeon medicolegally and allowing recording of any improvement after successful realignment.

## RESULTS

The index procedure was carried out on eight patients (five males and three females). The average age was 12.8 years (6-18 years). The mean follow-up was 5.25 months (2-11 months). The diagnoses were SCFE (*n* = 4), Perthes disease (*n* = 2), osteoid osteoma (*n* = 1) and osteochondroma of the neck of the femur (*n* = 1) [Table 1]. The children with SCFE underwent capital femoral realignment in two and osteoplasty in two; osteoplasty was performed in the two children with Perthes disease, with additional elevation of head and bone grafting in one of them. Excision of the tumor was carried out in the children with osteochondroma and osteoid osteoma.

The amplitude of pressure recorded varied between the patients; however, all five vascular heads showed a wave form corresponding to the ECG trace. In two patients with acute SCFE, no pressure was recordable initially; however, after capital realignment, the vascularity was restored in one after repositioning of the head and observed introperatively. In both the children with acute SCFE, a bone scan was performed post-operatively, which showed an intact vascularity in one and avascularity of the femoral head in the other. In the two children with healed SCFE, there was a good wave form and pressure. These children underwent osteoplasty for femoro-acetabular impingement. In the child with residual Perthes disease, the pressure recorded was 2 mm Hg and, his preoperative and postoperative radiographs had evidence of avascularity. In one child with modified Elizabeth Town Stage 2 A Perthes disease, the wave form was absent and the pressure was 0. He underwent fusion of the growth plate, osteoplasty, curetting of the avascular anterolateral segment and bone grafting, the latter two surgical steps being performed to improve vascularity. His radiographs also showed avascular changes as expected. The details are described in Table 1.

**Table 1:** Clinical details and observations (intraoperative and followup)

Age/sex	Diagnosis	Surgical procedure	Femoral head pressure mm/hg	Wave form	Postoperative vascularity by bone scan	Radiological evidence of AVN preop	Postop Radiological evidence of AVN	Followup in months
12 M	Acute on chronic SCFE	Capital realignment + surgical dislocation	0	Absent	Affected epiphysis decreased tracer activity	Not seen	AVN seen in the last follow up	7
10 F	Acute unstable SCFE	Capital realignment + surgical dislocation	40	Present	Symmetrical uptake both hips	Not seen	Not seen	7
14 F	Healed SCFE	Osteoplasty	44	Present	Not done	Not done	Not done	7
14 M	Healed SCFE	Osteoplasty	39	Present	Not done	Not done	Not done	2
14 F	Perthes (residual)	Osteoplasty	2	Absent	Not done	Present	Present	2
15 M	Perthes stage 2A	Osteoplasty+bone graft+ fusion of growth plate	0 (Head), 17 (Trochanter)	Absent	Not done	Present	Present	11
6 M	Osteoid osteoma posterior femoral neck	Excision osteoid osteoma	40	Present	Not done	Absent	Not seen	4
18 M	Diaphseal aclasis osteochondroma neck femur	Excision osteochondroma + osteoplasty	52	Present	Not done	Absent	Not seen	2

M: Male, F: Female, SCFE: Slipped capital femoral epiphysis, AVN: Avascular necrosis, Preop: Preoperative

**Figure 3:**
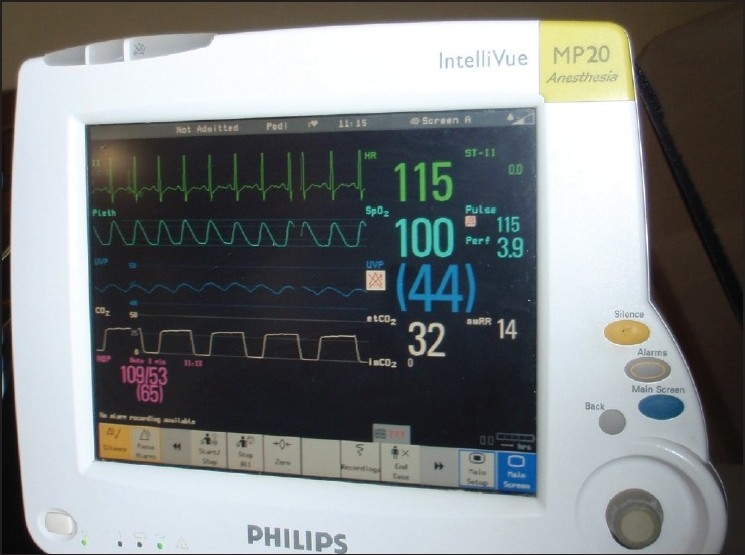
The wave form and the trace from the arterial transducer (blue) connected to the epidural catheter in the femoral head is synchronous with the ECG trace (green)

**Figure 4:**
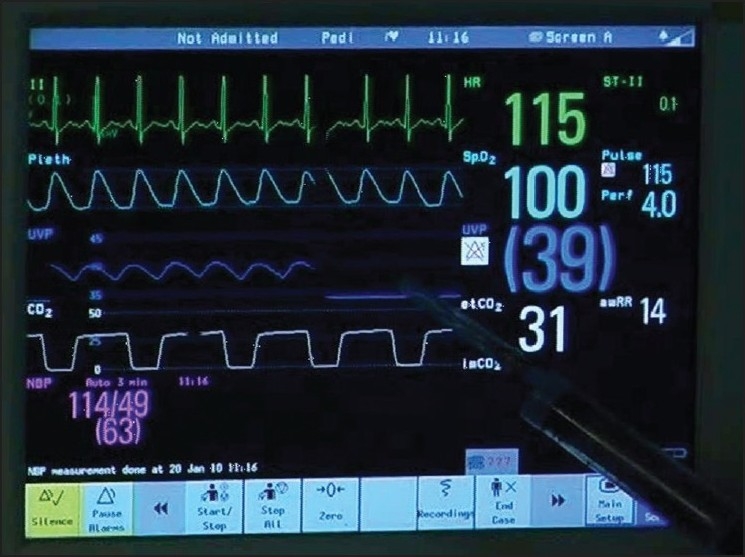
Pressure on the vascular pedicle at the base of the neck posteriorly causes the wave form to disappear (marked by needle of syringe) indicating blood flow compromise to the femoral head

**Figure 5:**
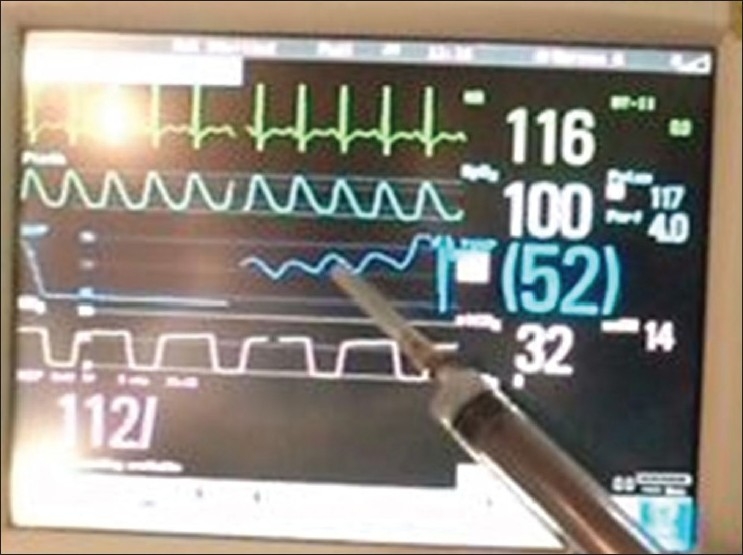
On removing the digital compression from the vascular pedicle at the base of the femoral neck, the wave form re-appears, indicating restoration of blood flow

The technique applied in a 14-year-old undergoing a safe surgical dislocation procedure for osteoplasty in a slipped capital femoral epiphysis showed a mean pressure of 44 mm Hg and a wave form that corresponded to the ECG trace. The trace shown on the monitor represented the femoral head pressure [Figure 3]. We also found that the wave form disappeared when the vascular pedicle was compressed at the base of the neck posteriorly [Figure 4], only to reappearwhen the compression was relieved [Figure 5].

In the remaining two children with osteoid osteoma and osteochondroma, respectively, the monitoring of the head pressure and wave form was done while excising the posterior neck lesions, and the vascualrity stayed intact during the procedure. Their radiographs on follow-up so far have shown no avascular necrosis.

The costing of the device when using an epidural catheter is approximately 2000 rupees, excluding the anesthesia monitor. It is possible to further reduce the cost by using an IV canula instead of the epidural catheter for a single attempt at measurement.

## DISCUSSION

The measurement of intraosseous pressure of the femoral head for functional studies of the femoral head vascularity has been described by Ficat and Arlet.^6^ They used a trocar introduced into the marrow and connected to a simple manometer to assess the femoral head pressure. They also demonstrated the relationship of the wave pattern of the pressure with the ECG trace and carotid artery pulsations, and found the pressure measured by their technique to be constant at 20 mm Hg and 5 mm higher than the trochanteric pressure in adults. These investigations were performed under local anesthesia.

Our measurements of the pressure using an arterial transducer in the anesthetized patient undergoing an osteoplasty using safe surgical dislocation by the Ganz approach showed wave forms corresponding to the QRS complex on ECG tracing and to the arterial pulsation. We also found that the wave form disappeared when the vascular pedicle was compressed at the base of the neck posteriorly [Figure 4], only to reappear when the compression was relieved [Figure 5]. The higher pressures obtained when compared with Ficat and Arlet’s observations are probably due to differences in the site of catheter insertion, equipment used, age group of the patient, position of femoral head and anesthesia.^7^

The absolute pressures may not be of much significance in the determination of bone blood flow in surgical situations. However, the wave form and its relationship to ECG and arterial flow (pulse oxymeter reading on the anesthesia monitor) is important for determining the arterial supply to the part of the head being assessed.

This simple and effective technique is an easily accessible tool for an orthopedic surgeon to assess the hip vascularity, with a wide range of applications in the field of trauma and hip reconstructive surgery. This will also have use in replacement surgery, such as surface replacement arthroplasty, where an on-table assessment of suitability of the posterior head neck area is essential to prevent a future failure due to collapse of an avascular segment.

## CONCLUSION

The assessment of the blood flow of the femoral head is an established technique with many indications. The present technique describes an easy cheap alternative using equipment available in most operating rooms with the anesthetist. The system is extremely simple to assemble and use.
